# Memory and Consciousness—Usually in Tandem but Sometimes Apart

**DOI:** 10.1097/WNN.0000000000000337

**Published:** 2023-03-23

**Authors:** Christopher R. Madan

**Affiliations:** School of Psychology, University of Nottingham, Nottingham, United Kingdom

**Keywords:** episodic memory, consciousness, nonhuman animals, mental time travel, hippocampal replay

## Abstract

Episodic memory, the ability to remember specific events from one’s personal past, has been the subject of research for several decades, with a particular emphasis on its relationship with consciousness. In the December 2022 issue of *Cognitive and Behavioral Neurology*, Budson, Richman, and Kensinger shed new light on this complex topic with a comprehensive exploration of consciousness. In this commentary, I present three propositions about the relationship between episodic memory and consciousness: (1) Episodic memory is usually associated with conscious retrieval; (2) it is possible to have consciousness without episodic memory; and (3) episodic memory can be accessed without conscious retrieval. Drawing from studies conducted with nonhuman animals, I provide evidence to support each of these propositions and discuss how they relate to the theory presented by Budson et al (2000). Although some of my propositions differ from their views, their work has been valuable in stimulating ongoing discussions to advance our understanding of memory.

I applaud Budson, Richman, and Kensinger (2022) on their comprehensive exploration of consciousness in the December 2022 issue of *Cognitive and Behavioral Neurology*. They discuss many topics in their article—including a breadth of cognitive functions, from perception to free will, along with implications to our understanding of a multitude of clinical disorders and nonhuman animals. I also appreciate the inclusion of the supplemental material, as this provided additional situational insight into the development of their framework. Here, I discuss only a subset of the topics presented in their article, focusing primarily on the implications to our understanding of episodic memory.

Although the relationship between memory and consciousness is a key topic in Budson et al (2022), I found some specific details about this relationship to be unclear. Here, I make three specific propositions and discuss both how they fit into Budson and colleagues’ presented framework and my own perspective and relevant evidence.

1. Episodic memory is *usually* associated with conscious retrieval.
2. It is possible to have consciousness *without* episodic memory.
3. Episodic memory can be accessed *without* conscious retrieval.

Some authors have suggested that nonhuman animals have the capability for episodic memory based on neurobiology, specifically, the involvement of the hippocampus (Allen and Fortin, 2013; Barbosa and Castelo-Branco, 2022; Crystal, 2018, 2021; DeVito and Eichenbaum, 2010). These perspectives are aligned with my present view in suggesting that episodic memory is not uniquely human, but they do not map onto this article’s presented propositions directly. For additional context, in humans, the hippocampus appears to be particularly important when integrating or separating information, which is an operation that is necessary for episodic memory (Cowell et al, 2019; Madan, 2023; Mayes et al, 2007; Ranganath and Ritchey, 2012).

## Episodic Memory is Usually Associated With Conscious Retrieval

The only aspect of this proposition that is contentious is the inclusion of the word *usually*. The current established definition of episodic memory, as proposed by Tulving (2002, p 5), is:

*Episodic memory is a recently evolved, late-developing, and early-deteriorating past-oriented memory system, more vulnerable than other memory systems to neuronal dysfunction, and probably unique to humans. It makes possible mental time travel through subjective time, from the present to the past, thus allowing one to re-experience, through autonoetic awareness, one’s own previous experiences. Its operations require, but go beyond, the semantic memory system. Retrieving information from episodic memory (remembering or conscious recollection) is contingent on the establishment of a special mental set, dubbed episodic “retrieval mode.” Episodic memory is subserved by a widely distributed network of cortical and subcortical brain regions that overlaps with but also extends beyond the networks subserving other memory systems. The essence of episodic memory lies in the conjunction of three concepts—self, autonoetic awareness, and subjectively sensed time.*

It is worth highlighting that Tulving developed this definition over several decades. Munsat (1967) was the first to use the term *episodic memory*; Quillian (1966) was the first to use the term *semantic memory.* Tulving (1972) adopted both of these terms and put them within the same framework (Tulving, 1983b, p 29). That said, a distinction between *memory for events* and *memory for knowledge* has been discussed by many authors throughout history (Madan, 2023; Rubin and Umanath, 2015). Tulving (1985) later added the requirement of consciousness to episodic memory. While I have previously attempted to dissociate episodic memory from consciousness (Madan, 2020), reading Budson and colleagues’ 2022 work along with recent collaborative discussions has provided me with more insight into consciousness (Tsikandilakis et al, 2021) and memory phenomenology (Dev et al, 2022; Wardell et al, 2021).

One of the key features of episodic memory that is thought to be unique to humans is *mental time travel*, or the ability to consciously re-experience a past episode (Roberts, 2002; Suddendorf and Busby, 2003; Tulving, 2002). It could be argued that neural recordings demonstrating hippocampal replay could represent mental time travel. Critically, evidence of replay has consistently been shown in nonhuman animals (Diba and Buzsáki, 2007; O’Neill et al, 2010; Skaggs and McNaughton, 1996).

Providing additional behavioral evidence of mental time travel and replay, some studies have shown that auditory cues that are present during learning can be presented again during slow-wave sleep to reactivate the learning experience—a procedure known as *targeted memory reactivation* or *cued memory retrieval*. While the work with humans is more well known (Hu et al, 2020; Rasch et al, 2007), this phenomenon was first shown with rats (Hars and Hennevin, 1987) and has continued in more recent work (Bendor and Wilson, 2012).

Before moving to further propositions, it is worth reminding readers that there is a specific term that was coined back in 1904 to describe the process of conscious episodic memory retrieval—*ecphory* (Schacter et al, 1978; Semon, 1904). Tulving (1983a) provided further insight into ecphory, describing memory traces as multidimensional, with a subjective recollective experience as one of the components.

## It Is Possible to Have Consciousness Without Episodic Memory

It is well accepted that consciousness is difficult to measure (Armstrong, 1898; Birch 2022; Carruthers et al, 2020; Mellor, 2019; Sandberg et al, 2010). Moreover, it is well established that consciousness is not simply present or absent but varies along a continuum. Hunt et al (2022) provided an overview of approaches for measuring consciousness; a variety of clinical disorders are associated with altered states of consciousness.

Of particular relevance, authors have found that individuals who had lost the ability to acquire new episodic memories—such as H.M. and Clive Wearing—were still conscious despite their memory deficits (Gregory et al, 2014; Sacks, 1985; Scoville and Milner, 1957; Wilson and Wearing, 1995). Budson and colleagues (2022, p 279) agree with this proposition: “Consciousness, at least in the ordinary sense of the term, does not appear to be disrupted by damage to the hippocampus and related structures. Individuals with such damage can certainly consciously experience many perceptions, decisions, and actions.”

## Episodic Memory can be Accessed *Without* Conscious Retrieval

This proposition is the crux of my commentary—the earlier two propositions are uncontentious, and I only included them in order to lay the foundation for this dissociation between episodic memory and consciousness. The nonconscious access of episodic memory is briefly described by Budson and colleagues (2022, p 272) as being possible: “It is possible that they still do engage the episodic memory system, but only partially, and not strongly enough for a full, true, conscious episodic memory to be formed.” However, this is immediately after described as “no true episodic memory.”

For many decades, studies of episodic memory in nonhuman animals have used the term *episodic-like* when demonstrating findings that appeared to show episodic memory while still conceding to Tulving’s definition (Clayton et al, 2001; Fugazza et al, 2016; Pahl et al, 2007). However, more recently, some researchers have dropped this caveat and discuss episodic memory directly (Crystal, 2018, 2021; Madan, 2023; Panoz-Brown et al, 2016).

A key issue with our current operationalization of episodic memory is that it typically cannot be assessed using solely objective criteria. That said, some authors have suggested such criteria, allowing it to be consistently assessed in both humans and nonhumans (Antunes and Biala, 2012; Barbosa and Castelo-Branco, 2022; Crystal, 2021; Easton and Eacott, 2010). More specifically, the test of episodic memory here would correspond to the memory of *what, where, and when*—that is, the memory of a specific episode. This change in conceptualization bears some commonalities with the recent shift in characterizing fear conditioning in nonhuman animals instead as threat conditioning (LeDoux, 2012, 2014), which also relates to memory systems, developing objective definitions, and evaluating the inferences we can make about consciousness in other species.

Budson and colleagues (2022, p 282) believe that all mammals are conscious and that they vary in the complexity of their conscious experience. The main claim of these authors is that episodic memory requires and necessitates consciousness. In contrast, I distinguish between the capability of consciousness and its complexity from the moment-to-moment contents of conscious experience. Here, I suggest *conscious memory recall* as a subset of the episodic memory system—not as a synonym.

This change to only require what–where–when memory, without necessitating subjective recollection, would be a departure from the conventional definition of episodic memory. Those authors who claim that nonhuman animals do not have episodic memory would agree that some animals *can* demonstrate what–where–when memory specificity (Suddendorf and Busby, 2003). The possibility of nonconscious episodic memory could be one account for preferences as memory—where rapid, one-shot learning experiences can affect decision-making (Mason et al, 2022; Palombo et al, 2021; Weber and Johnson, 2006).

## CONCLUSION

In the December issue of *Cognitive and Behavioral Neurology*, Budson and colleagues (2022) presented a comprehensive discussion of consciousness and proposed it as an underlying basis for episodic memory. I found some of these views contentious and so here I presented three specific propositions for an updated view of episodic memory. I also summarized the alignment of these propositions with Budson and colleagues and the wider literature. A broader consensus view of episodic memory is still needed, but ongoing dialogues are making important progress.
